# Decreased ovarian function and autophagy gene methylation in aging rats

**DOI:** 10.1186/s13048-020-0615-0

**Published:** 2020-02-03

**Authors:** Qiuyuan Li, Minghui Cai, Jiao Wang, Qiang Gao, Xiaocheng Guo, Xiaotong Jia, Shanshan Xu, Hui Zhu

**Affiliations:** Department of Physiology, Harbin Medical University, Harbin, 150086 China

**Keywords:** Ovarian aging, Autophagy, DNA methylation, DNA methyltransferase

## Abstract

**Background:**

Degeneration of ovarian function is an obvious feature of female aging. In addition, studies have shown that autophagy decreases with age, and DNA methylation is a hallmark epigenetic pattern during aging. However, it is not clear whether the expression and DNA methylation of autophagy genes are involved in the declines in ovarian function that occur during aging.

**Results:**

Three groups of rats were used: 6-month-old (6 M) rats, 12-month-old (12 M) rats and 24-month-old (24 M) rats. Serum E_2_ levels and the mRNA and protein expression levels of Atg5, Atg12, Atg16L, Beclin1 and Lc3B were significantly decreased in aged rats. In addition, the methylation levels of the *Atg5* gene were significantly increased in aged rats. The expression of the *Dnmt1* and *Dnmt2* genes decreased with aging; however, the expression of the *Dnmt3A* and *Dnmt3B* genes gradually increased with aging.

**Conclusions:**

Decreased autophagic activity was involved in the declines in ovarian function in aging rats. Upregulation of the DNA methyltransferases Dnmt3A and Dnmt3B may have led to methylation of the autophagy genes *Atg5* and *Lc3B* to ultimately cause the observed decreases in autophagic activity.

## Introduction

Aging is characterized by time-dependent declines in function resulting from progressive biochemical and physiological dysregulation [1]. Compared with other tissues, the ovaries are much more severely affected by aging. The numbers of follicles and the quality of oocytes in the ovaries decrease with age, resulting in a gradual decline in fertility [2, 3]. During the transition period from perimenopause to menopause, increases in follicle-stimulating hormone (FSH) levels and decreases in estrogen levels caused by ovarian aging can lead to considerable follicular atresia and to depletion of primordial follicles [4, 5]. Previous research has revealed that the number of primordial follicles decreases at a rate of approximately 1000 follicles per month during the reproductive years of life and that only approximately 1000 follicles remain in the ovaries during the final menstrual stage (menopause) [4].

Aging is a degenerative and irreversible biological process caused by interactions among multiple factors and organs. Autophagy is a process in which cells degrade damaged organelles and biological macromolecules in lysosomes. Autophagy is a necessary process for the maintenance of intracellular homeostasis under physiological conditions, but under some conditions, such as inflammatory responses, oxidative stress, and organelle aging, autophagy may be overactivated or inhibited [6, 7]. Therefore, the regulation of intracellular autophagic activity is very important for the maintenance of normal physiological functions [8, 9]. A large body of evidence indicates that autophagic activity declines with age [10, 11]. Toth et al. has reported that loss of function of *Atg1, Atg7, Atg18* and *Beclin1* decreases lifespan in the nematode *Caenorhabditis elegans*. Komatsu et al. has reported that loss of *Atg7* leads to neurodegeneration and died within 28 weeks of birth [12–14]. Tan et al. has reported that remarkable autophagy-related changes, including the accumulation of ubiquitin-positive proteins and decreases in autophagic activity, occur with age in the brains of senescence-accelerated mouse prone 8 (SAMP8) mice [15]. Abdellatif et al. has reported that cardiomyocyte-specific *Atg5* ablation in aged C57BL/6 mice dramatically accelerates declines in cardiac function, resulting in shortened lifespan [16].

DNA methylation is a hallmark epigenetic pattern that occurs during aging, but aging-associated DNA methylation is not stochastic [17]. DNA methyltransferases (Dnmts) play vital roles in DNA methylation. Mammalian Dnmts are usually classified as Dnmt1, Dnmt2 or Dnmt3 (which includes four subtypes: A, B, C and L). Notably, Dnmts have different functions during the methylation process [17, 18]. Dnmt1, which is required for the maintenance of all methylation in the genome, can restore specific methylation patterns on daughter strands in accordance with the patterns on parental DNA during replication [19]. Dnmt2 methylates small transfer RNAs, and Dnmt3A and Dnmt3B are responsible for establishing DNA methylation patterns [20, 21]. Dnmt3C can protect male germ cells from retrotransposon activity, which is essential for mouse fertility [19].

Previous studies have suggested that declines in autophagy might be associated with aging. However, it is not clear whether the expression and DNA methylation of autophagy genes are involved in the declines in ovarian function that occur during aging. In this study, the expression and methylation levels of autophagy-related genes in aging rats were studied.

## Results

### Age-dependent declines in ovarian function

**Fig. 1:**
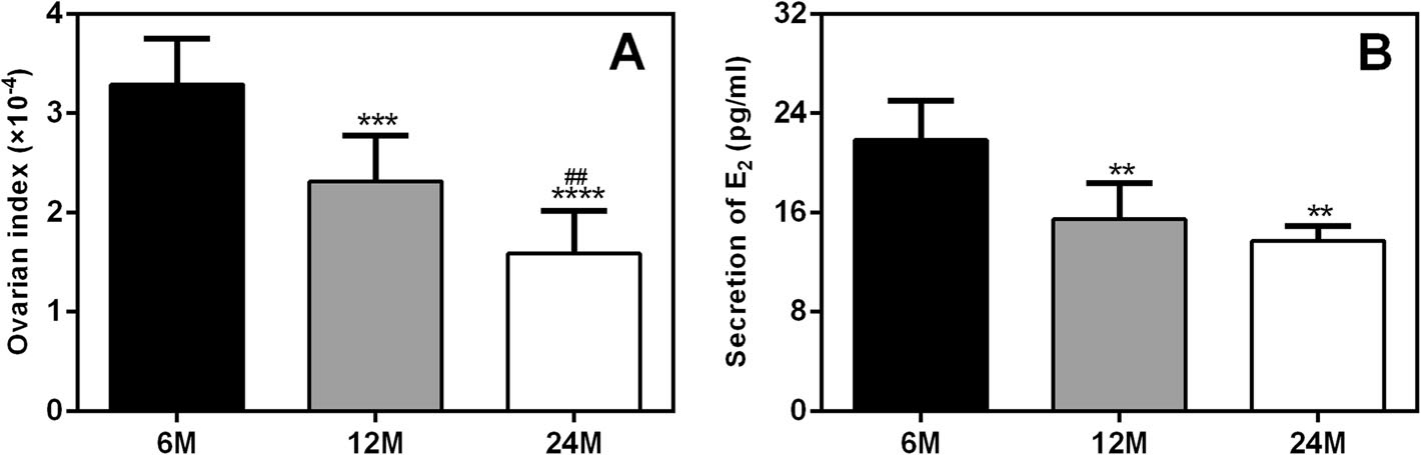
Age-dependent declines in the ovarian index and E_2_ secretion in rats. (**a**) Ovarian index (ratio of ovarian weight to body weight). (**b**) Secretion of E_2_. E_2_: estradiol. The ovarian index values and the serum E_2_ levels of 12 M and 24 M rats were significantly lower than those of 6 M rats. 6 M: 6-month-old, 12 M:12-month-old, 24 M:24-month-old. Compared with 6 M rats, **P < 0.01,***P < 0.001,****P < 0.0001; compared with 12 M rats, ^##^*P* < 0.01

In this study, the ovaries of rats were weighed, and the ovarian index (ovarian weight/body weight) values were calculated. The results showed that ovary weight decreased with aging: the ovarian index values of 12-month-old (12 M) and 24-month-old (24 M) rats were significantly lower than those of 6-month-old (6 M) rats (Fig. 1a). In addition, the serum estradiol (E_2_) levels in rats were measured by radioimmunoassay (RIA). The results showed that the serum E_2_ levels in 12 M and 24 M rats were significantly lower than those in 6 M rats (Fig. 1b). These results confirm that the endocrine functions of the ovaries present age-dependent declines.

### Age-dependent mRNA expression of autophagy-related genes in the ovaries of rats

**Fig. 2:**
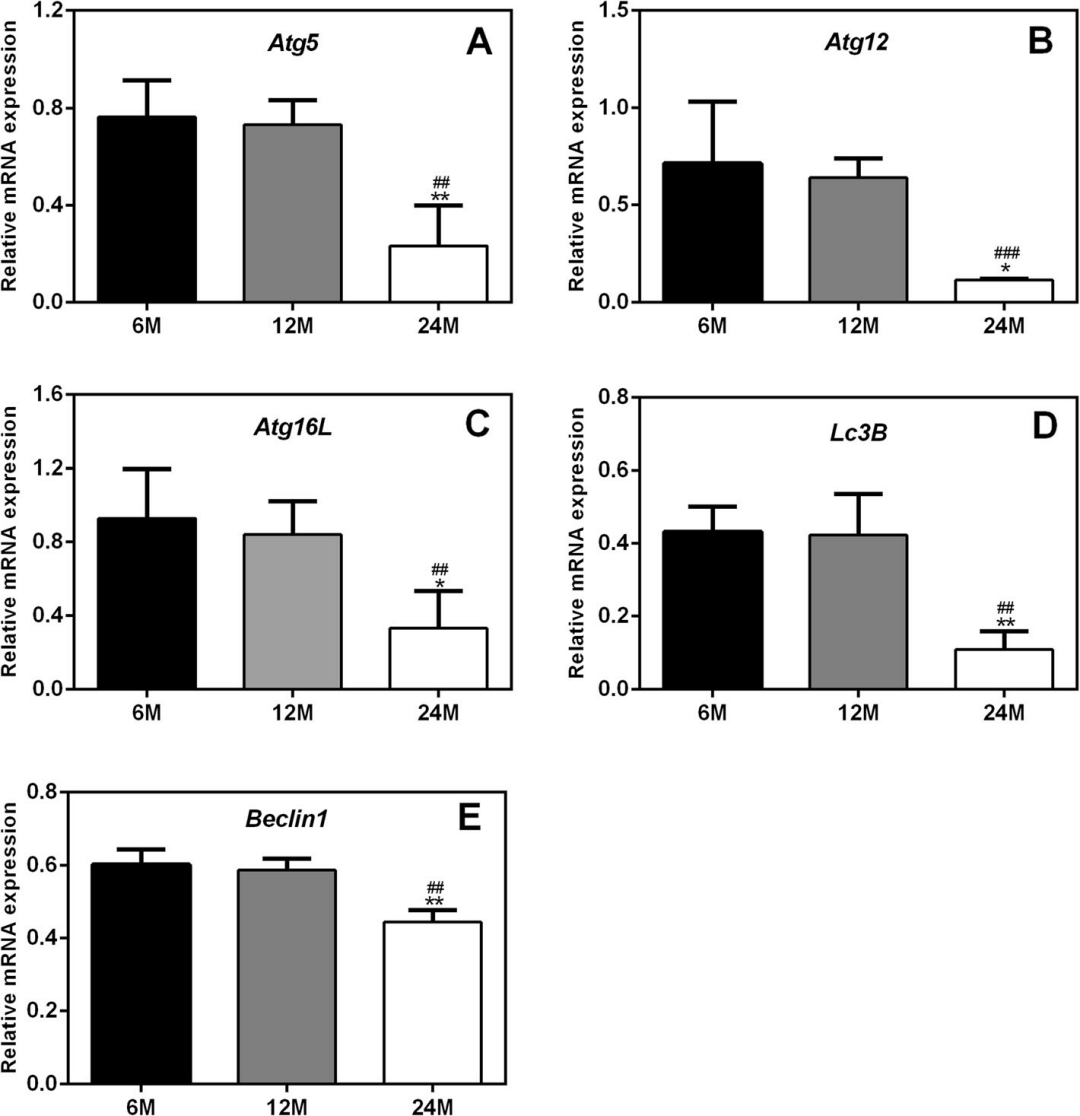
mRNA expression of autophagy-related genes in the ovaries of rats. (**a**-**e**) mRNA expression levels of *Atg5*, *Atg12*, *Atg16L*, *Lc3B* and *Beclin1.*Compared to those in 6 M and 12 M rats, the mRNA expression levels of *Atg5*, *Atg12*, *Atg16L*, *Beclin1* and *Lc3B* in 24 M rats were significantly decreased. 6 M: 6-month-old, 12 M: 12-month-old, 24 M: 24-month-old. Compared with 6 M rats, *P < 0.05, **P < 0.01; compared with 12 M rats, ^##^*P* < 0.01, ^###^*P* < 0.001

Quantitative real-time PCR was used to analyze the mRNA expression of autophagy-related genes, including *Atg5*, *Atg12*, *Atg16L*, *Beclin1* and *Lc3B.* As shown in Fig. 2, compared to those in 6 M and 12 M rats, the mRNA expression levels of *Atg5*, *Atg12*, *Atg16L*, *Beclin1* and *Lc3B* in 24 M rats were significantly decreased. However, there were no significant differences in the mRNA expression of any autophagy-related genes between 12 M and 6 M rats. These results suggest that ovarian autophagic function is decreased in aged rats.

### Age-dependent protein expression of autophagy-related genes in the ovaries of rats

**Fig. 3:**
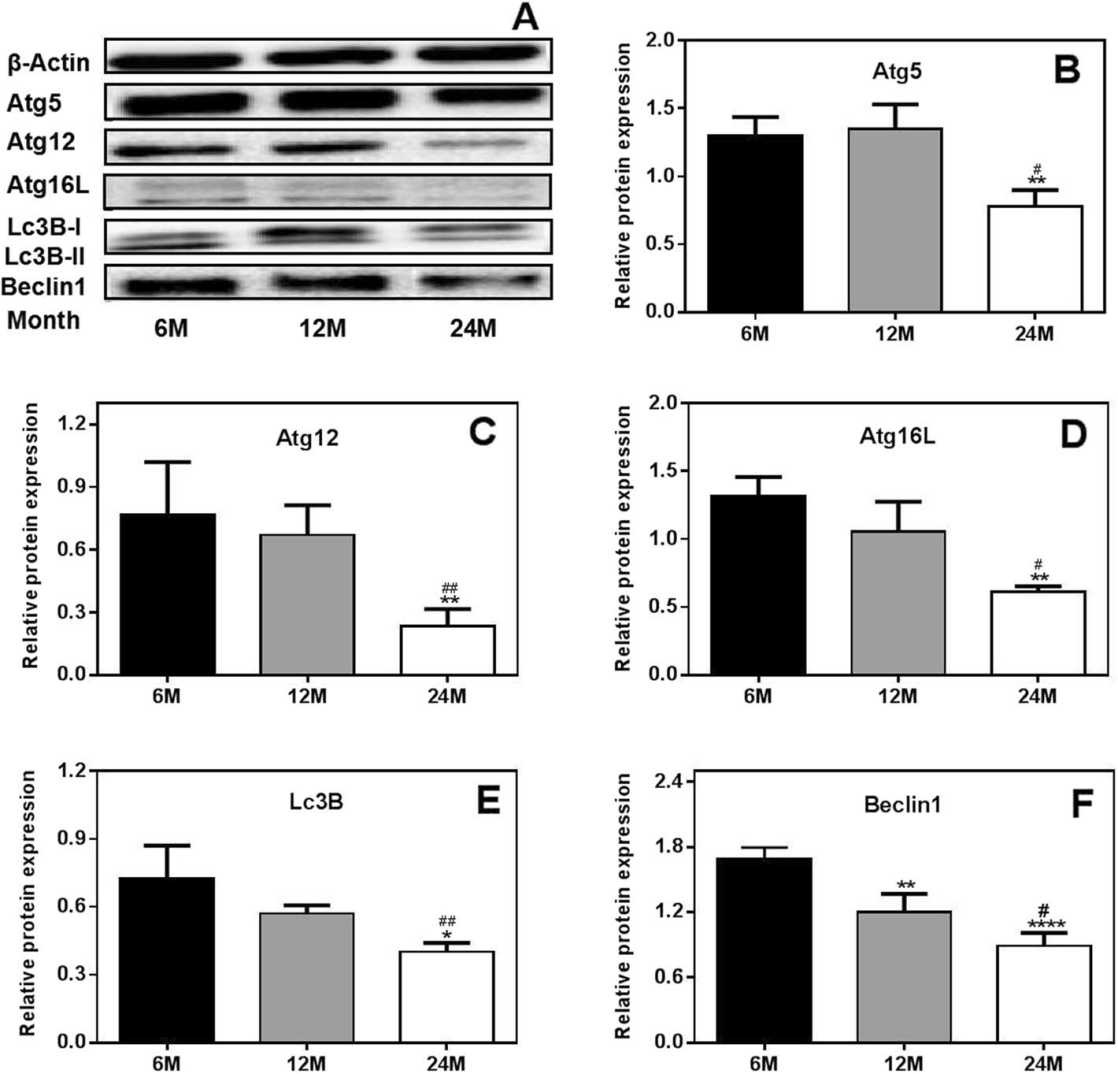
Protein expression of autophagy-related genes in the ovaries of rats. (**a**) Western blot results. (**b**-**f**) Protein expression levels of Atg5, Atg12, Atg16L, Lc3B and Beclin1. Protein expression levels of Atg5, Atg12, Atg16L, Beclin1 and Lc3B were significantly lower in 24 M rats than in 6 M and 12 M rats. 6 M: 6-month-old, 12 M: 12-month-old, 24 M: 24-month-old. Compared with 6 M rats, *P < 0.05, **P < 0.01, ****P < 0.0001; compared with 12 M rats, ^#^*P* < 0.05, ^##^*P* < 0.01

To further investigate the expression of autophagy-related genes in rat ovaries, the protein expression levels of Atg5, Atg12, Atg16L, Lc3B and Beclin1 were detected using Western blot analysis. The results showed that the protein expression levels of Atg5, Atg12, Atg16L, Beclin1 and Lc3B were significantly lower in 24 M rats than in 6 M and 12 M rats. There were no significant differences in the expression levels of autophagy-related genes between the 12 M rats and the 6 M rats (Fig. 3). These results further confirm that age-dependent reductions in autophagy occur in rat ovaries.

### Age-dependent DNA methylation is correlated with age in the ovaries of rats

**Fig. 4:**
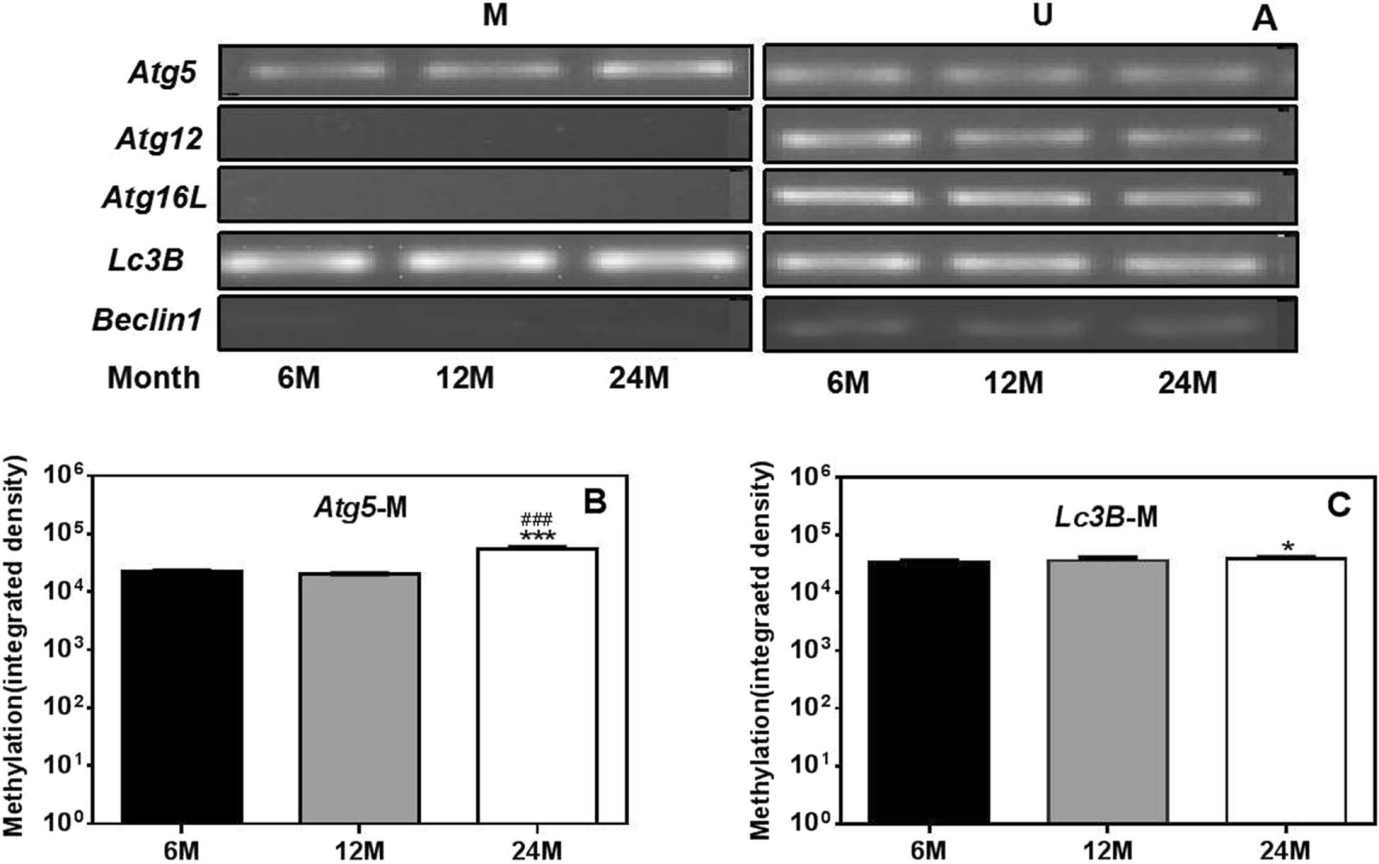
Age-dependent DNA methylation of autophagy-related genes in the ovaries of rats. (**a**) Agarose gel electrophoresis of DNA methylation. (**b**-**c**) Quantitative methylation levels of *Atg5* and *Lc3B.* .*Atg5* gene and *Lc3B* gene methylation levels were significantly higher in the ovaries of 24 M rats than in those of 6 M rat. 6 M: 6-month-old, 12 M: 12-month-old, 24 M: 24-month-old, M: methylated bands, U: unmethylated bands. Compared with 6 M rats, *P < 0.05, ***P < 0.001; compared with 12 M rats, ^###^*P* < 0.001

Based on the results of real-time PCR and Western blot, we found that the expression of autophagy-related genes in the ovaries of rats decreased with age. To further investigate the mechanism of the decreases in autophagic function, we detected the methylation statuses of the promoter regions of the autophagy-related genes *Atg5*, *Atg12*, *Atg16L*, *Beclin1* and *Lc3B* in the ovaries of rats by methylation-specific PCR (MSP). The results are shown in Fig. 4. We found that the autophagy genes *Atg5* and *Lc3B* were methylated in rat ovaries and that *Atg5* gene methylation levels were significantly higher in the ovaries of 24 M rats than in those of 6 M and 12 M rats. In addition, the methylation levels of the *Lc3B* gene were significantly greater in the ovaries of 24 M rats than in those of 6 M rats. However, methylation of the *Atg12*, *Atg16L* and *Beclin1* genes was not detected in the rat ovaries. These results suggest that the decreases in *Atg5* and *Lc3B* gene expression in the ovaries of aged rats might be related to gene methylation (Fig. 4).

### Age-dependent changes in Dnmts in the ovaries of rats

**Fig. 5:**
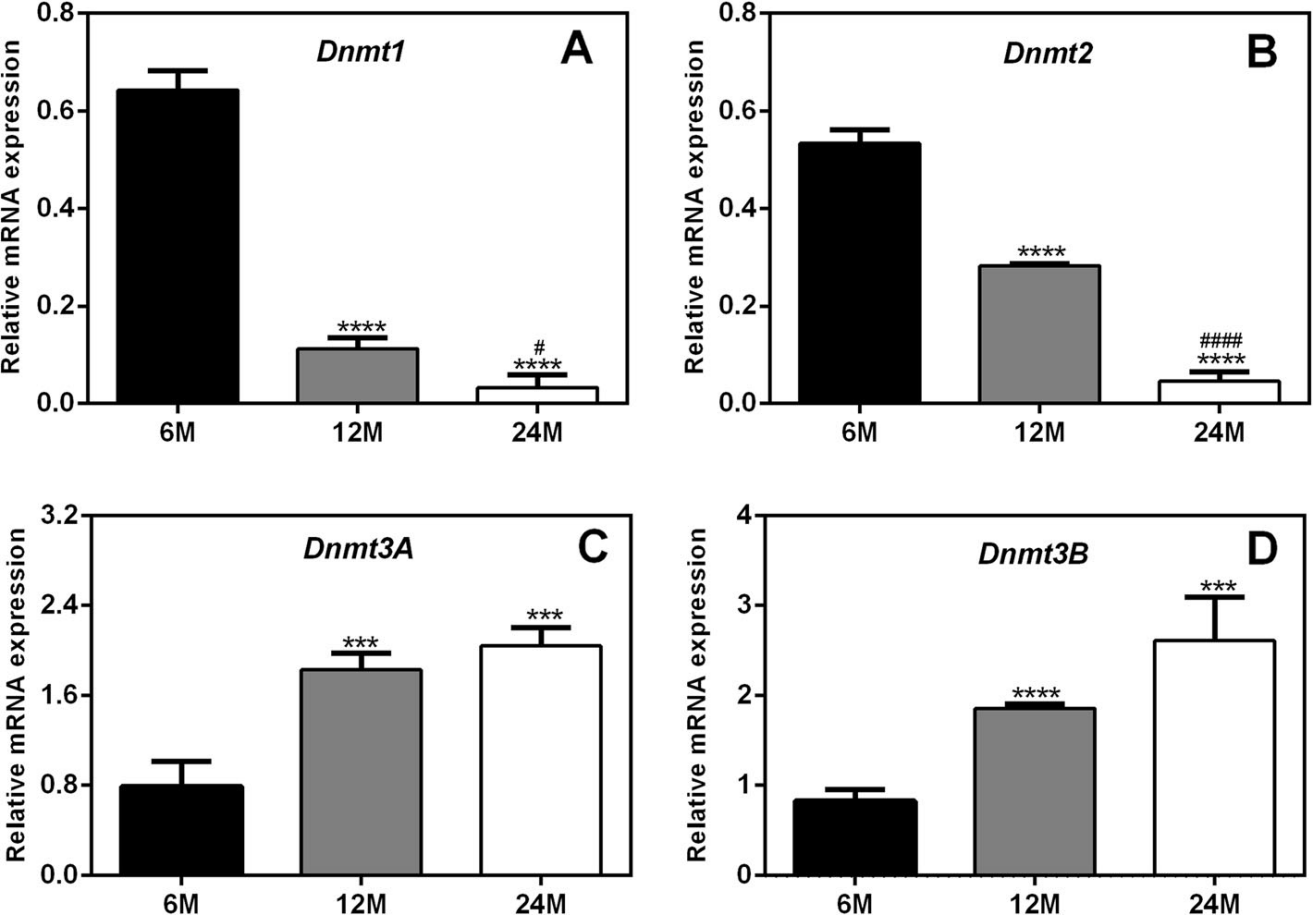
mRNA expression levels of *Dnmt1*, *Dnmt2, Dnmt3A* and *Dnmt3B* in the ovaries of rats. Compared to those in 6 M rats, the expression levels of the *Dnmt1* and *Dnmt2* genes in 12 M and 24 M rats were significantly decreased. However, the expression levels of the *Dnmt3A* and *Dnmt3B* genes gradually increased with aging. 6 M: 6-month-old, 12 M: 12-month-old, 24 M: 24-month-old. Compared with 6 M rats, ***P < 0.001,****P < 0.0001; compared with 12 M rats, ^#^*P* < 0.05, ^####^*P* < 0.0001

The MSP results showed that the methylation levels of the autophagy genes *Atg5* and *Lc3B* were increased in aged rats. Because Dnmts play important roles in gene methylation, the expression levels of *Dnmt1*, *Dnmt2*, *Dnmt3A* and *Dnmt3B* in the ovaries of rats were detected by real-time PCR. As shown in Fig. 5, compared to those in 6 M rats, the expression levels of the *Dnmt1* and *Dnmt2* genes in 12 M and 24 M rats were significantly decreased. However, the expression levels of the *Dnmt3A* and *Dnmt3B* genes gradually increased with aging.

**Fig. 6:**
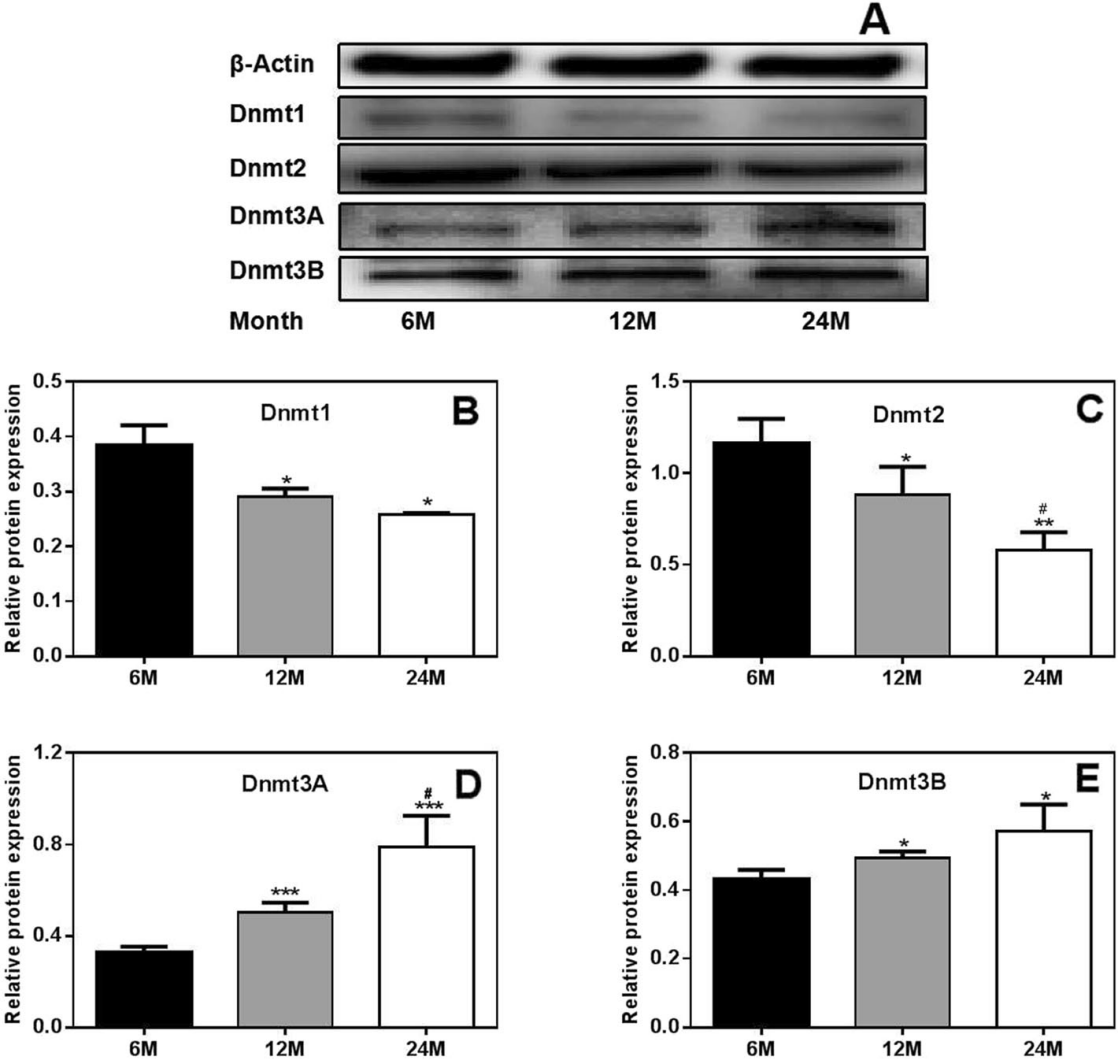
Protein expression of Dnmt1, Dnmt2, Dnmt3A and Dnmt3B in the ovaries of rats.(**a**) Western blot results. (**b**-**e**) Protein expression levels of Dnmt1, Dnmt2, Dnmt3A and Dnmt3B. Compared to those in 6 M rats, the protein expression levels of Dnmt1 and Dnmt2 in 12 M and 24 M rats were decreased. However, the protein expression of Dnmt3A and Dnmt3B was greater in 12 M and 24 M rats than in 6 M rats. 6 M: 6-month-old, 12 M: 12-month-old, 24 M: 24-month-old. Compared with 6 M rats, *P < 0.05,***P < 0.001; compared with 12 M rats, ^#^*P* < 0.05

The protein expression of Dnmt1, Dnmt2, Dnmt3A and Dnmt3B in the ovaries of rats was further detected by Western blot analysis. The results showed that the changes in protein expression were consistent with, albeit less significant than, the observed changes in mRNA expression. Compared to those in 6 M rats, the protein expression levels of Dnmt1 and Dnmt2 in 12 M and 24 M rats were decreased. However, the protein expression of Dnmt3A and Dnmt3B was greater in 12 M and 24 M rats than in 6 M rats. Moreover, the changes in Dnmt2 and Dnmt3A protein expression were more significant in 24 M rats than in 12 M rats (Fig. 6).

Based on the above results, we hypothesize that the increases in Dnmt3A and Dnmt3B, but not Dnmt1 and Dnmt2, might be related to increases in the methylation of the autophagy genes *Atg5* and *Lc3B* in aged rats.

## Discussion

The aging process of ovaries is characterized by morphological atrophy and functional declines in reproductive hormones (especially estrogen). The rat is the most widely used model animal in studies on ovarian function. Rats exhibit periodic follicular development and sex hormone changes in a process called the estrous cycle, which is similar to the human menstrual cycle [5, 22]. The secretion of ovarian hormones decreases in an age-dependent manner in rats; Acuna et al. found that the estrogen levels of 8- to 14-month-old rats are significantly lower than those of 6 M rats [23]. In this study, we measured ovarian weight and estrogen levels in 6 M, 12 M and 24 M rats. The results showed that with increasing age, ovary weight decreased gradually, and serum E_2_ levels decreased significantly. Consistent with our results, Nie et al. reported that the ovaries of aged (40-week-old) female C57BL/6 mice exhibit structural and functional deterioration and that secretion of E_2_ significantly decreases with consecutive superovulations in these naturally age-deteriorated ovaries [24].

Autophagy is widely involved in biological processes such as growth, development, apoptosis and aging [25, 26]. The autophagy-related genes *Atg*, *Beclin1* and *Lc3B* are the core molecules that maintain and induce autophagy [27]. Different *Atg* genes participate in different steps of autophagy [28]. For example, activation of *Atg1* promotes the initiation of autophagosome formation [29]; the *Atg12* conjugation system (*Atg12*-*Atg5*-*Atg16L* complex) is necessary for autophagosome formation, phagophore elongation and cargo recognition [30, 31]; and *Atg7* is an essential catalyst for autophagosome assembly. The conversion of Lc3B-I to Lc3B-II is also necessary for autophagosome formation [32]. It has been established that autophagy in various tissues decreases gradually with age and that autophagy progression is closely related to the expression of various autophagy-related genes [14].

Therefore, in this study, we detected the expression of autophagy-related molecules at the gene and protein levels. We found that the mRNA and protein expression levels of Atg5, Atg12, Atg16L, Beclin1 and Lc3B were significantly decreased in aged rats, suggesting that ovarian aging in these rats was accompanied by decreases in autophagic function. Khlil et al. observed that the mRNA and protein expression levels of Atg5 and Lc3B were significantly reduced in macrophages from aged mice [33]. In addition, Glatigny et al. noted decreased accumulation of the Lc3B-II, Beclin1 and Atg5 proteins in the hippocampi of 16-month-old mice [34]. In another study, decreased expression levels of *Lc3B* and *Beclin1* were observed in the kidneys of aged rats [35]. Based on the above results, we suspect that ovarian aging in rats was accompanied by reductions in autophagic activity.

Gene expression levels are negatively correlated with DNA methylation levels [36, 37]. Stubbs et al. found considerable CpG methylation in the cerebral cortices, lungs, hearts and other tissues of aged mice [37]. In this study, we found DNA methylation changes associated with the *Lc3B* and *Atg5* genes among all the downregulated autophagy-related genes in the ovaries of aged rats. Thus, we speculate that the decreases in autophagy gene expression in the ovaries of aged rats may be at least partly due to DNA methylation of some of these genes.

DNA methylation, or the addition of a methyl group onto DNA, is catalyzed by Dnmts, including Dnmt1, Dnmt2, and Dnmt3 (Dnmt3A and Dnmt3B). Dysregulation of DNA methylation has been implicated in diseases including neurological diseases, inflammatory diseases and cancers [38, 39]. In this study, we found that both the mRNA and protein expression levels of Dnmt3A and Dnmt3B were significantly increased in aged rats and that the increased expression of these genes might be related to increased methylation of the autophagy genes *Atg5* and *Lc3B*. We also found that the expression of Dnmt1 and Dnmt2 in rat ovaries gradually decreased with aging; however, these decreases were not found to be related to the methylation of autophagy-related genes, indicating that Dnmt1 and Dnmt2 may regulate ovarian aging through other mechanisms. Previous studies have shown that Dnmt1 can regulate the functions of various types of stem cells, including embryonic stem cells and osteoblasts [40, 41]. We speculate that the role of Dnmt1 in aging may be related to the proliferation and dysfunction of stem cells [42]. The findings of several published studies are consistent with our data. For example, Fasolino et al. observed that *Dnmt3A* expression significantly increases in the striatum throughout aging in a mouse model of aging-related changes in DNA methylation [43]. In addition, Casillas et al. found that while *Dnmt1* expression declines during aging, *Dnmt3B* expression increases during aging and in neoplastically transformed human fibroblasts [44]. Furthermore, Zhou et al. confirmed that the mRNA and protein expression levels of Dnmt3A and Dnmt3B are significantly elevated and that those of Dnmt1 are significantly downregulated in oligodendrocyte precursor cells from 16-month-old rats [45].

## Conclusion

In this study, we found that decreased autophagic activity was involved in the declines in ovarian function in aging rats. Upregulation of the Dnmt3A and Dnmt3B may have led to methylation of the autophagy genes *Atg5* and *Lc3B* to ultimately cause the observed decreases in autophagic activity.

## Methods

### Animals and sample collection

Female Sprague-Dawley (SD) rats were obtained from the experimental animal center of Harbin Medical University. The rats were divided into three groups (*n* = 8/group): the 6-month-old (6 M, adult) rat group, the 12-month-old (12 M, menopausal) rat group, and the 24-month-old (24 M, aged) rat group. The rats were anesthetized with 10% chloral hydrate, serum samples were obtained by centrifuging blood samples at 3000 rpm for 15 min at 4 °C, and the ovaries were either embedded in paraffin for histological analysis or frozen in liquid nitrogen and stored at − 80 °C for molecular analysis. The protocols for all animal experiments were approved by the Institutional Animal Care and Use Committee (IACUC) of Harbin Medical University.

### Experimental plan

Female SD rats were divided into three groups (*n* = 8/group): the 6 M, 12 M and 24 M groups. Serum was collected and used for measurement of the hormone estradiol (E_2_) by radioimmunoassay (RIA). The mRNA and protein expression of autophagy genes in ovarian tissue was examined by real-time PCR and Western blot analysis*.* The DNA methyltransferases and methylation levels of autophagy genes were examined by real-time PCR and methylation-specific PCR (MSP).

### Hormone detection

The levels of E_2_ in serum were detected using commercial RIA kits (Sino-UK Institute of Biological Technology, Beijing, China).

### Western blot analysis

The protein expression levels of autophagy-related genes were determined by Western blot analysis. Proteins were extracted from ovaries using RIPA lysis buffer, and the protein concentrations were determined by BCA protein assay. Total protein (20 μg) was electrophoresed on 10% or 12% SDS-PAGE gels and transferred onto PVDF membranes (Immobilon-P, Millipore, USA). The membranes were blocked for 2 h in 5% milk/TBST and then incubated overnight at 4 °C with primary antibodies. β-Actin (1:6000), Atg5 (1:1000), Lc3B (1:1000), Atg16L (1:1000), Atg12 (1:1000) and Beclin1 (1:1000) primary antibodies were purchased from Cell Signaling Technology; Dnmt1 (1:250), Dnmt3A(1:250) and Dnmt3B (1:500) primary antibodies were purchased from Affinity Biosciences; and a Dnmt2 (1:500) primary antibody was purchased from Santa Cruz.. The following day, the membranes were incubated with horseradish peroxidase (HRP)-conjugated goat anti-rabbit or goat anti-mouse IgG (Santa Cruz, USA) for 1 h at room temperature. The blot bands were visualized using Pierce ECL Western Blot Substrate (Engreen Biosystem, China). β-Actin was used as the endogenous control and the relative band density was quantified by densitometry using ImageJ software.

### Real-time PCR

The mRNA expression levels of autophagy-related genes were determined by real-time PCR (Applied Biosystems 7500). Total RNA was extracted from ovaries using TRIzol reagent (Invitrogen, Carlsbad, CA, USA). First-strand cDNA was synthesized from the total RNA using a PrimeScript 1st Strand cDNA Synthesis Kit (Takara Bio Inc., China). The PCR products were amplified using a SYBR Premix Ex TaqII Kit (Takara Bio Inc., China) in 20 μL mixtures containing 1 μL of cDNA template and 0.5 μM forward and reverse primers (Table 1). *β-Actin* was used as the endogenous control, and the data were evaluated by the 2-ΔΔCt method.

**Table 1:** Sequences of the Primers Used in Real-Time PCR

Gene	Forward primer	Reverse primer
*Atg5*	CACTGGGACTTCTGCTCCTG	TTCTTCAACCAAGCCAAACC
*Atg12*	AAACGAAGAAATGGGCTGTG	GAAGGGGCAAAGGACTGATT
*Atg16L*	CTGTGCTTTTCCCGTCTTTC	GCCCTGATTTGGTTTCCAC
*Lc3B*	GGTGTTTTTCTCCTGGTTTGG	GCACTTGGACTTCAGCCTTC
*Beclin1*	GCCTCTGAAACTGGACACG	CCTCTTCCTCCTGGCTCTCT
*Dnmt1*	CGACGACGCTAAGGACGATGATG	GCCTTGTTGCTCGCCTCTGTC
*Dnmt2*	GCGGTTGCGAGAGGATGGAAC	ACGTCAATAGCAGCCACCACATG
*Dnmt3B*	AGACCAGAGGCCGCAGATCAAG	TCCGCTTCACCATCTCCATCTCC
*Dnmt3A*	TGCCAGTCATCCGCCACCTC	CTCCGTCCTCTCGTTCTTGGTG

### MSP

The methylation levels of the promoter regions of autophagy-related genes were assessed using MSP. Genomic DNA was extracted from ovaries using a MiniBEST Universal Genomic DNA Extraction Kit (Takara Bio Inc., China) and then treated with an EZ DNA Methylation-Gold Kit (Zymo Research, USA) for bisulfite conversion. The converted DNA was used as a template for MSP amplification with specific primers (Table 2) designed by Methylprimer online (http://www.urogene.org/methprimer/indexl.html). The MSP products were analyzed on 3% agarose gels, and quantitative analyses were performed using ImageJ software.

**Table 2:** Sequences of the Primers Used in Methylation-Specific PCR

Gene	Forward primer	Reverse primer
*Atg5*-M	GTTGGTTTAAGAAGGAAATTAGCG	CCCGAACTACTCCTAAACGTA
*Atg5*-U	GGTTTAAGAAGGAAATTAGTGGAT	ACAAAACCCAAACTACTCCTAAACATA
*Atg12*-M	ATAGATTAGTTAGGGCGAGTAGCG	CCCGACTATTTCCTATCGAA
*Atg12*-U	AGATTAGTTAGGGTGAGTAGTGAGT	TCAACCCAACTATTTCCTATCAA
*Atg16L*-M	GTAAGGAAATAGATATTAGCGGAT	GACCGAAAACGTCGTAAAAC
*Atg16L*-U	AAGTAGTAAGGAAATAGATATTAGTGGAT	AAACTCAACCAAAAACATCATAAAAC
*Lc3B*-M	GGAGATATATAAGGGAAGTGATCGTC	GACGCTATTTAAAAATCTTCTCG
*Lc3B*-U	AGGAGATATATAAGGGAAGTGATTGTT	CCAACACTATTTAAAAATCTTCTCA
*Beclin1*-M	TTTATAAGAGAGTATGGACGGTTTC	CCGATCGACTAACTAAAAACTTC
*Beclin1*-U	AATTTATAAGAGAGTATGGATGGTTTT	CCAATCAACTAACTAAAAACTTCC

### Statistics

All data in this study are expressed as the mean ± standard deviation. Statistical calculations were performed by one-way ANOVA using GraphPad Prism version 6.0 (GraphPad Software Inc.). *P* < 0.05 was considered to indicate a statistically significant difference.

## Data Availability

Not applicable.

## Abbreviations

12 M: 12-month-old
24 M: 24-month-old
6 M: 6-month-old
Dnmt1: DNA methyltransferases1
Dnmt2: DNA methyltransferases 2
Dnmt3A: DNA methyltransferases 3A
Dnmt3B: DNA methyltransferases 3B
Dnmts: DNA methyltransferases
E_2_: Estradiol
FSH: Follicle-stimulating hormone
MSP: Methylation-specific PCR
